# Study on Impact of Monomers Towards High Molecular Weight Bio-Based Poly(ethylene Furanoate) via Solid State Polymerization Technique

**DOI:** 10.3390/polym16233305

**Published:** 2024-11-26

**Authors:** Johan Stanley, Eleftheria Xanthopoulou, Margaritis Kostoglou, Lidija Fras Zemljič, Dimitra A. Lambropoulou, Dimitrios N. Bikiaris

**Affiliations:** 1 Laboratory of Chemistry and Technology of Polymers and Colors, Department of Chemistry, Aristotle University of Thessaloniki, GR-541 24 Thessaloniki, Greece; johansta@chem.auth.gr (J.S.); exanthoa@chem.auth.gr (E.X.); 2 Laboratory of Chemical and Environmental Technology, Department of Chemistry, Aristotle University of Thessaloniki, GR-541 24 Thessaloniki, Greece; kostoglu@chem.auth.gr; 3 Faculty of Mechanical Engineering, University of Maribor, SI-2000 Maribor, Slovenia; lidija.fras@um.si; 4 Laboratory of Environmental Pollution Control, Department of Chemistry, Aristotle University of Thessaloniki, GR-541 24 Thessaloniki, Greece; dlambro@chem.auth.gr; 5 Center for Interdisciplinary Research and Innovation (CIRI-AUTH), Balkan Center, GR-570 01 Thessaloniki, Greece

**Keywords:** bio-based polymers, 2,5-furan dicarboxylic acid, Dimethyl 2,5-furan dicarboxylate, poly(ethylene 2,5-furan dicarboxylate), poly(ethylene furanoate), solid state polymerization, thermal properties

## Abstract

In recent years, bio-based poly(ethylene furanoate) has gained the attention of packaging industries owing to its remarkable properties as a promising alternative to fossil-based polymers. It is necessary to synthesize high-molecular-weight polymers using effective and straightforward techniques for their commercialization. In this present work, poly(ethylene 2,5-furan dicarboxylate) (PEF) was produced with a high molecular weight of 0.43 dL/g using 2,5-furan dicarboxylic acid (FDCA) or its derivative Dimethyl-2,5-Furan dicarboxylate (DMFD), followed by solid-state polymerization (SSP) conducted at different temperatures and reaction times. The intrinsic viscosity ([*η*]), carboxyl end-group concentration (–COOH), and thermal properties of the produced polyesters were evaluated using differential scanning calorimetry (DSC). The results indicated that the SSP process improved the melting temperature and crystallinity of both the PEF samples as the reaction times and temperatures increased, as corroborated by DSC and X-ray diffraction (XRD) analyses. Additionally, both intrinsic viscosity and number-average molecular weight saw an increase with longer SSP durations and higher temperatures, while the concentration of carboxyl end groups decreased, aligning with expectations. The overall results indicate that PEF (DMFD) samples exhibited a significant increase in crystallization and molecular weight, attributed to their lower degree of crystallinity and their monomer’s high purity.

## 1. Introduction

The bio-based market is currently dominated by drop-in bio-based polymers (from renewable raw materials with comparable material properties to their fossil counterparts; e.g., bio-based poly(ethylene terephthalate) (PET)) and non-biodegradable polymers [1,2]. Other than the resistance to biodegradability, greenhouse gas (GHG) emissions constitute the major issue forcing the chemical industry to shift from petroleum-derived plastics towards bio-based, benign polymers. Poly(ethylene 2,5-furandicarboxylate) (PEF) is a bio-based polymer with a striking ability to reduce GHG emissions and promising properties to replace fossil-based PET in packaging industries. A recent study conducted by Eerhart et al. on life cycle assessment (LCA) of corn-starch-based PEF shows a reduction in GHG emission by about 45–55% (with fossil-based ethylene glycol (EG)) or by 68–82% (with bio-based EG) relative to fossil-based PET [3]. Due to its low GHG emissions, PEF gains increasing attention from the chemical industries and governmental agencies as an alternative to conventional polymers [4]. Avantium (Amsterdam, Netherlands) has already started to produce PEF on a pilot scale aiming to operate the world’s first 2,5-furan dicarboxylic acid (FDCA) flagship plant in 2024 to produce 5000 tons of FDCA per annum (5 kta), which is the key building block for the PEF polyester [5].

PEF is a 100% recyclable bio-based polyester with better barrier, mechanical, and thermal characteristics than PET, making it ideal for demanding applications like food packaging, bottles, and fibers [6,7,8]. For example, the automotive, electrical, and electronics sectors can take advantage of enhanced PEF-based thermoplastics due to their outstanding mechanical characteristics, ability to be remolded or reshaped, thermal stability, and dimensional consistency. Additionally, it is possible to develop sustainable, recyclable, and durable adhesives modified with PEF [9,10]. Due to the rigidity and polarity of its furan ring, it exhibits lower O_2_, CO_2_, and H_2_O permeability compared to PET. PEF exhibits similar mechanical properties to PET, with a slightly higher elastic modulus and smaller elongation at break [11]. The thermal stability of PEF is slightly lower than PET, but it is stable until 350 °C [12]. A recent study by Pluta et al. aimed to investigate the thermal behavior of PEF in relation to varying cold-crystallization temperatures. The study concluded that the cold-crystallization temperature significantly influences the morphology and thermal properties of PEF [13]. Van der Mass et al. demonstrated a novel method for producing high-molecular-weight PEF polyesters using diguaiacyl oxalate (DGO) as a traceless chain extender, which allows for the coupling of lower molecular weight polymer chains and eliminating the need for metal catalysts [14].

Solid-state polymerization (SSP) is a highly efficient technique for synthesizing high-molecular-weight polymers suitable for various industrial applications, such as bottles, films, and fibers. It offers an optimized approach for achieving high production efficiency and consistent product quality, as evidenced in the polyester industry. This process stands out for its low energy requirements and solvent-free nature, reducing environmental impact and eliminating the need for solvent recovery systems, making it a greener alternative to solution polymerization [15]. Additionally, SSP is cost-effective due to its straightforward process design and the absence of costly catalysts often used in other polymerization methods. Operating at lower temperatures than melt polymerization, SSP minimizes energy consumption and reduces the risk of side reactions that could compromise polymer quality. Its versatility allows for its application across a wide range of polymers, including biodegradable polymers, thermoplastics, and liquid crystalline polymers, making it suitable for various industrial uses [16].

Knoop et al. studied the preparation of high-molecular-weight furan-based polyesters using a combination of melt polycondensation and solid-state polycondensation techniques. Dimethyl furan-2,5-dicarboxylate (DMFD) was chosen as a starting monomer due to its purity after two recrystallization cycles and less color formation. A tenfold increase in the *Mw* (83,000 g/mol) of PEF was observed after 72 h of SSP, which was sufficiently high for bottle and fiber application [17]. Hong et al. have managed to synthesize PEF with 0.60 dL/g intrinsic viscosity [*ղ*] using DMFD as the starting monomer via a melt polycondensation technique. In order to expand the usage of PEF for bottles and packaging applications, the SSP technique was applied. After 48 h of SSP, a moderate increase in intrinsic viscosity of 0.72 dL/g was observed [18]. Gabirondo et al. conducted an interesting study on the depolymerization of commercial PEF and further repolymerization. The repolymerization was conducted using melt polycondensation and further SSP was carried out up to 48 h. The successful formation of biopolymers with similar chemical and thermal properties like virgin PEF offers a sustainable end-of-life option for PEF [19].

Our research group also conducted various studies on SSP to improve the molecular weight of PEF. Kasmi et al. aimed to synthesize high-molecular-weight PEF polyesters using DMFD as the starting monomer, catalyzed by tetrabutyl titanate (TBT) and followed by SSP carried out at different reaction times and temperatures. Following SSP, the intrinsic viscosity increased significantly, exceeding 1 dL/g after just 5 h of reaction at 205 °C, demonstrating its suitability for industrial manufacturing processes [20]. Papadopoulos et al. have made a comparative study between poly(propylene furanoate) (PPF) and poly(butylene furanoate) (PBF), which were synthesized via two-step melt polycondensation using DMFD as starting monomers, followed by SSP performed at different temperatures and reaction times [21].

Our previous work was focused on a comparative study of synthesizing high-molecular-weight poly(ethylene furanoate) using FDCA or its derivative DMFD as the starting monomer for food packaging applications. Our research group observed that FDCA promoted the production of high-molecular-weight PEF [22]. Although much research has been carried out to improve the molecular weight of furan-based polyesters using different monomers, catalysts, and nanocomposites, to the best of our knowledge, no comparative study has been performed regarding the effect of monomer type on improving the molecular weight of PEF by using melt polycondensation followed by the SSP technique. Thus, in this work, PEF was synthesized using both FDCA and DMFD as starting monomers using the melt polycondensation technique and subjected to the SSP technique for further improving the molecular weight of the PEF. Our aim was to see which monomer gives the higher molecular weight increase during SSP. The effect of temperature and time on the increase of molecular weight of both PEF (FDCA) and PEF (DMFD) samples was monitored, while complementary data were provided by means of XRD and DSC.

## 2. Materials and Methods

### 2.1. Materials

2,5-Furan dicarboxylic acid (BioFDCA X000230-2003) was purchased from Corbion (Gorinchem, The Netherlands). Diethylene glycol (DEG) (anhydrous, 99.8%), titanium butoxide, and antimony trioxide were purchased from Aldrich Co. (London, UK). DMFD was synthesized using FDCA and methanol. All other reagents and solvents utilized in the process were of analytical grade.

### 2.2. Synthesis of PEF Using Dimethyl 2,5-Furandicarboxylate (DMFD)

The PEF polyesters were synthesized in a glass batch reactor through a two-stage melt polycondensation process consisting of transesterification and polycondensation steps. A 1:1.5 molar ratio of DMFD and diethylene glycol (DEG) was used, with titanium butoxide (TBT) (400 ppm) serving as the catalyst. To eliminate air, the reactor containing the reagents was evacuated and purged with nitrogen multiple times before commencing the reaction.

In the transesterification step, the reaction mixture was heated at 160–190 °C for 4 h under a nitrogen atmosphere while being continuously stirred at 200 rpm. Nearly all the theoretical methanol produced during the reaction was removed via distillation, marking the completion of this step.

For the polycondensation step, a vacuum of 5.0 Pa was gradually applied over 15 min to prevent excessive foaming, reduce oligomer sublimation, and remove excess diols. The reaction was then carried out at 220–240 °C for 4 h to complete the polycondensation process. The reaction mixture was continuously stirred at 100–150 rpm under vacuum conditions. Upon completion of the polycondensation reaction, the resulting polyesters were extracted, milled, and subsequently washed with methanol [22].

### 2.3. Synthesis of PEF Using 2,5-Furandicarboxylic Acid (FDCA)

In the initial esterification step, FDCA and DEG were combined in a 1:2.1 molar ratio. The flask was evacuated and purged with nitrogen three times to ensure the complete removal of air. The reaction mixture was preheated to 170 °C for 30 min, followed by heating at 190–210 °C for 2 h while stirring at 200 rpm under a nitrogen atmosphere. Generally, within the first 1–1.5 h of the first step, EG distillation occurs. After 2.5 h, the oligomers were removed with a heating pistol.

Antimony trioxide (Sb_2_O_3_) (300 ppm) was charged into the polymerization reactor as a catalyst before the polycondensation step. A vacuum (5.0 Pa) was applied slowly for 15 min to begin the polycondensation process. The vacuum was applied slowly to avoid evacuating reaction solutions from the flask. The increase in the temperature took place at the same time as the vacuum. Then, the temperature increased to 250 °C for 4 h, and 260 °C for 2 h. At the same time, the stirring speed decreased (100-70-50 rpm) to avoid high shear stress. Finally, the samples were retrieved from the reaction mixture, washed with methanol, milled, and characterized [22].

### 2.4. Solid State Polymerization

The SSP process was carried out using an apparatus that included four test tubes linked to a vacuum line and placed in a thermostatically controlled salt bath. About 1.5 g of milled PEF polyester was added to each glass tube while maintaining a vacuum of 3–4 Pa. The reaction was conducted at steady temperatures of 185 °C, 190 °C, and 195 °C for PEF polyesters made from both FDCA and its derivative DMFD monomers. The test tubes were taken out of the bath at intervals of 1, 2, 4, and 6 h, and the resulting polymers were analyzed for intrinsic viscosity ([*η*]), carboxyl end-group content, and thermal characteristics.

### 2.5. Characterization

#### 2.5.1. Intrinsic Viscosity

The intrinsic viscosity of the materials was determined using an Ubbelohde viscometer (Schott Gerate GmbH, Hofheim, Germany) at 25 °C. The samples were dissolved in a 60/40 (*w*/*w*) solution of phenol and tetrachloroethane by heating the mixture at 80 °C for 10 min. After cooling, the samples were filtered to remove any solid particles. The intrinsic viscosity ([*η*]) was then calculated using the Solomon–Ciuta Equation (1):

(1) $$\left[\eta \right]=\frac{{[2\{\frac{t}{t_{0}}-\ln\left(\frac{t}{t_{0}}\right)-1\}]}^{\frac{1}{2}}}{c}$$

where c is the concentration of the solution, t_0_ is the flow time of pure solvent, and t is the flow time of solution.

The number-average molecular weight ($\bar{M_{n}}$) of the samples was determined using Equation (2) following the Berkowitz equation [22]:

(2) $$\bar{M_{n}}=3.29\times 10^{4}\;{\left[\eta \right]}^{1.54}$$

#### 2.5.2. End-Group Analysis

The content of carboxyl end groups in the synthesized polyesters was determined by employing a titration method in a mixture of benzyl alcohol and chloroform, in accordance with Pohl’s approach [23]. The titration was conducted using a standard NaOH solution dissolved in benzyl alcohol, with phenol red serving as the indicator. This titration procedure was repeated three times, and the mean value of [COOH] was computed.

#### 2.5.3. Differential Scanning Calorimetry (DSC)

A PerkinElmer Pyris DSC-6 differential scanning calorimeter calibrated using pure indium and zinc standards was employed for the thermal analysis. Samples weighing 6–8 mg were sealed in aluminum pans, and all measurements were conducted under a nitrogen atmosphere with a flow rate of 20 mL/min. The PEF (FDCA) and PEF (DMFD) samples were first heated from 30 °C to 210 °C with a heating rate of 20 °C/min. The glass transition temperature (*T_g_*), melting temperature (*T_m_*), cold crystallization temperature (*T_cc_*), and enthalpies (*∆H_m_* and *∆H_cc_*) of the PEF (FDCA) and PEF (DMFD) samples were determined from these scans. Crystallinity degree (*X_c_^a^*) was calculated with Equation (3):

(3) $$X_{c^{a}}(\%)=\left(\frac{{\Delta H}_{m}-{\Delta H}_{cc}}{{\Delta H}_{m}^{0}}\right)\times 100$$

where *ΔH*^0^_*m*_ *=* 137 J/g, the heat of melting of the 100% crystalline PEF.

#### 2.5.4. Wide Angle X-Ray Diffraction Patterns (WAXRD)

X-ray powder diffraction (XRD) patterns were obtained using a MiniFlex II XRD system (Rigaku Co., Tokyo, Japan) with Cu Ka radiation (0.154 nm). All materials were analyzed at 2θ range of 5° to 50° with a scan speed of 1°/min. The % crystallinity was calculated from the XRD graphs through origin software (OriginPro 2024b-Learning edition) using Equation (4):


(4) $$X_{c^{b}}(\%)={\left(1+\frac{A_{am}}{A_{c}}\right)}^{-1}$$

A_am_ is the area of the amorphous halo, and A_c_ is the area of the crystalline peaks.

## 3. Modeling of the Solid-State Polymerization of PEF Polyesters Kinetics

### 3.1. Reaction Mechanism

The main reaction mechanisms occurring during the solid-state polymerization (SSP) of PEF polyesters include polycondensation/transesterification, esterification, thermal degradation, and the polycondensation of vinyl end groups [24]. The reaction mechanism is detailed in Equations (5)–(8), where k_1_ and K_1_ denote the forward and equilibrium rate constants for the transesterification process (Equation (5)), while k_2_ and K_2_ correspond to the rate constants associated with the esterification process (Equation (6)). The kinetic rate constants for the degradation and polycondensation of reactions involving vinyl end groups, regarded as one-way processes, are represented as k_d_ and k_v_ in Equations (7) and (8), respectively.

Polycondensation/transesterification


(5)

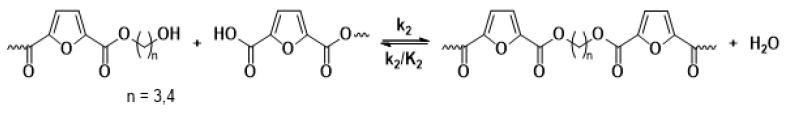



Esterification


(6)

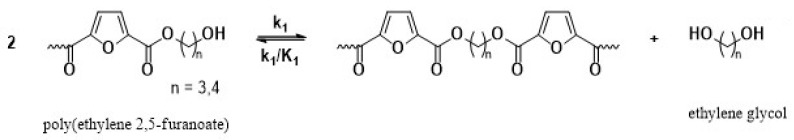



Thermal degradation


(7)

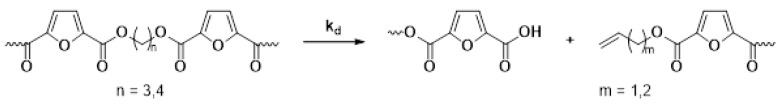



Polycondensation of vinyl end groups


(8)

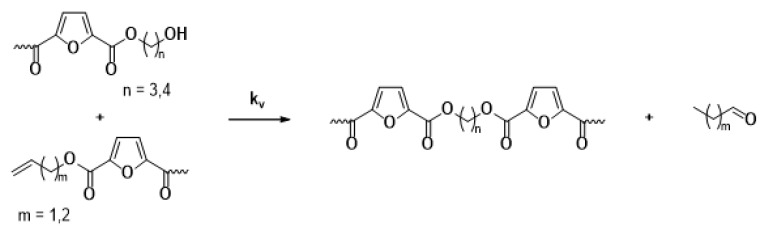



The molecular weight of the PEF polyesters was enhanced by means of three distinct reactions. In the first reaction, ethylene glycol was produced through the reaction of two hydroxyl end groups, as shown in Equation (5). In the second reaction, a carboxyl end group reacts with a hydroxyl group, producing water as a byproduct, as depicted in Equation (6). Additionally, a side reaction between a vinyl ester end group and a hydroxyl end group can release acetaldehyde, which also contributes to an increase in the molecular weight, as illustrated in Equation (8). On the other hand, when thermal degradation occurs, an ester bond in the macromolecular chain cleaves, producing a vinyl ester end group and a carboxyl end group, which can reduce the molecular weight of the polyester, as illustrated in Equation (7). The overall reaction rate can be influenced by intrinsic reaction kinetics, changes in the polymer’s degree of crystallization, diffusional limitations of the reactive end groups, and the desorption of volatile byproducts such as water and glycol [20,25].

### 3.2. Simplified Mathematical Model

The modeling of SSP kinetics presents significant challenges because it requires consideration of chemical kinetics and diffusion phenomena to accurately depict the change in concentration of reactive species throughout the reaction process [24]. This results in additional variation based on the distance from the interface. Consequently, to represent the reaction, a model that includes two independent variables and a series of partial differential equations encompassing multiple diffusional, kinetic, and crystallization parameters is essential [26]. Using such complex models to simulate just a few experimental data points lacks physical relevance. In this study, only five data points were recorded at each experimental condition, leading to the adoption of a simpler kinetic model as proposed by Ma and co-workers [27,28]. While this model was initially developed for the SSP of PET, our research group has effectively applied it to model the SSP of pristine PEF and also for nanocomposites of PEF and PET [20,21,29].

The mathematical model was developed based on the following assumptions.

- The kinetic rate constants are found to be unaffected by the polymer chain lengths, focusing solely on the reactivity of the end groups.
- The glycols and water are efficiently removed from the reaction mixture through the application of a high vacuum (below 3–4 Pa), leading to the omission of the reverse reactions presented in Equations (5) and (6).
- Owing to the polycondensation process operating at comparatively low temperatures (185–195 °C), neither thermal degradation nor acetaldehyde formation are considered side reactions (Equations (7) and (8) are eliminated).
- Any diffusional limitations resulting from the desorption of volatile species are disregarded.

Once these simplifications are taken into account, the change in rates of hydroxyl [OH] and carboxyl [COOH] end groups can be represented by Equations (9) and (10) [27,28].

(9) $$\frac{d[OH{]}_{t}}{dt}=-2k_{1}[OH{]}_{t}^{2}-k_{2}[COOH{]}_{t}[OH{]}_{t}$$

(10) $$\frac{d[COOH{]}_{t}}{dt}=-k_{2}[COOH{]}_{t}[OH{]}_{t}$$

where [COOH]_t_ and [OH]_t_ denote the actual “true” carboxyl and hydroxyl end-group concentration, respectively.

Ma and co-workers [27,28] coined the term “actual hydroxyl and carboxyl end-groups” to explain the slowdown in SSP kinetics at high [η] values. As a result, it was determined that a portion of the hydroxyl and carboxyl end groups ([OH]_i_ and [COOH]_i_, respectively) had been rendered temporarily inactive, and the actual concentration of OH and COOH in Equations (9) and (10) can be written as:

(11) $$[OH{]}_{t}=\left[OH\right]-[OH{]}_{i}$$

(12) $$[COOH{]}_{t}=\left[COOH\right]-[COOH{]}_{i}$$

where [COOH], [OH], [COOH]_i_, and [OH]_i_ stand for the total and temporarily inactivated concentrations of the COOH and OH groups, respectively.

Moreover, the end-group concentration can be used to determine the number-average molecular weight:

(13) $$\bar{M_{n}}=\frac{2}{\left[COOH\right]+[OH]}$$

In conjunction with Equations (2) and (11)–(13), along with (9) and (10), these equations form a collection of ordinary differential equations that are solved numerically utilizing the varying step-size Runge–Kutta method. The outcomes of this approach reveal changes in the concentrations of –COOH and –OH end groups, as well as the intrinsic viscosity over time during SSP. By concurrently fitting the concentrations of [OH], [COOH], and [*η*] to the experimental data points based on time, four adjustable parameters—k_1_, k_2_, [OH]*_I_*, and [COOH]*_i_*—are estimated for each experimental condition.

## 4. Results and Discussion

### 4.1. Synthesis and Kinetic Study of PEF Polyesters After SSP

The SSP of both PEF (FDCA) and PEF (DMFD) polyesters was carried out at 185, 190, and 195 °C for 1, 2, 4, and 6 h under vacuum. The values of intrinsic viscosity [*ղ*] and number-average molecular weight ($\bar{M_{n}}$) for the PEF (FDCA) and PEF (DMFD) polyesters, which were produced after solid-state polymerization (SSP) at various temperatures and durations, are presented in Table 1. The number-average molecular weights ($\bar{M_{n}}$) were calculated experimentally, measuring the intrinsic viscosity [*ղ*] using Equation (2).

After SSP, the PEF polyesters showed an increase in average molecular weight with respect to the increase in SSP time and temperature. The hydroxyl end groups of macromolecular chains reacted more readily with carboxyl end groups at temperatures that are close to PEF’s melting point, joining the chains and increasing PEF’s molecular weight. Moreover, the diffusion of generated byproducts, like ethylene glycol and water, was substantially slower at low SSP temperatures. Another factor is the increase in crystallinity that slows the diffusion rate of by-products that are formed [20]. For these reasons, the SSP temperature was chosen close to PEF’s melting point from 185 °C to 195 °C. As anticipated, due to the esterification and transesterification reactions taking place, the carboxyl end groups consistently diminished with longer SSP durations at every temperature tested, leading to an observed rise in the molecular weight of PEF [30]. Both SSP time and temperature played an important role in increasing the PEF’s molecular weight.

, g/mol) of PEF (FDCA) and PEF (DMFD) polyesters obtained after SSP at different temperatures and times. The degree of polymerization (DP) appears in parentheses.

**Figure 1:**
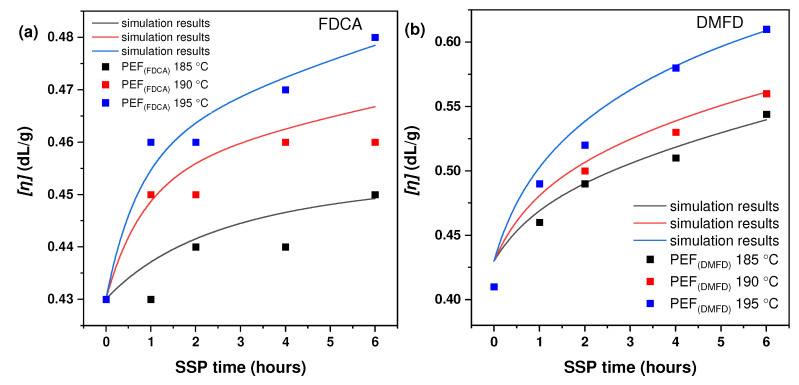
Variation of intrinsic viscosity [*ղ*] with respect to time (h) during SSP at different temperatures; (**a**) PEF (FDCA); (**b**) PEF (DMFD).

$\bar{M_{n}}$$\bar{M_{n}}$Figure 1a,b displays the increase in intrinsic viscosity values of synthesized PEF (FDCA) and PEF (DMFD) polyesters with respect to SSP time and temperatures. Although many research publications have been reported on the increase in the molecular weight of PEF polyesters, to overcome the inferior properties of PEF polyesters, to the best of our knowledge, no comparative study has been performed regarding the effect of monomers on improving the molecular weight of PEF. The initial intrinsic viscosity values of PEF (FDCA) and PEF (DMFD) polyesters were 0.43 dL/g and 0.41 dL/g, respectively. The intrinsic viscosity of the PEF (FDCA) sample was increased at a slow rate from 0.43 dL/g up to 0.45 dL/g at 185 °C after 6 h of SSP, up to 0.46 dL/g at 190 °C after 6 h of SSP, and up to 0.48 dL/g at 195 °C after 6 h of SSP.

The intrinsic viscosity of the PEF (DMFD) sample showed a higher increase from 0.41 dL/g up to 0.54 dL/g at 185 °C after 6 h of SSP, up to 0.56 dL/g at 190 °C after 6 h of SSP, and up to 0.61 dL/g at 195 °C after 6 h of SSP. It may be inferred that the PEF (DMFD) sample’s rapid growth in molecular weight was related to its lower degree of crystallinity since crystallinity has a significant impact on the mobility and diffusion rate of the polymer chain-end groups, which are found in the amorphous phase of semi-crystalline polyesters [21]. Also, the high purity of DMFD monomers obtained via esterification of FDCA with methanol under acidic conditions assisted in the rise of the molecular weight of the samples [31].

Also, as anticipated, raising the SSP reaction temperature from 185 to 195 °C, both transesterification and esterification were accelerated, which obviously favors the increase in intrinsic viscosity. The diffusion of the corresponding glycol and water controlled the corresponding reactions. The diffusion of byproducts was slower at low SSP temperatures. Increasing the SSP temperature close to the melting point of PEF polyesters, the carboxyl end groups of the polymer reacted more readily with hydroxyl end groups to form macromolecular chains, which raised the intrinsic viscosity of the polyester. Therefore, regardless of the type of polyester, intrinsic viscosity increased slowly at 185 to 195 °C [21,30]. It can be said that the [*ղ*] range achieved in this research was adequate to meet particular end-use necessities for food packaging, including beverage containers and sheets [20].

**Figure 2:**
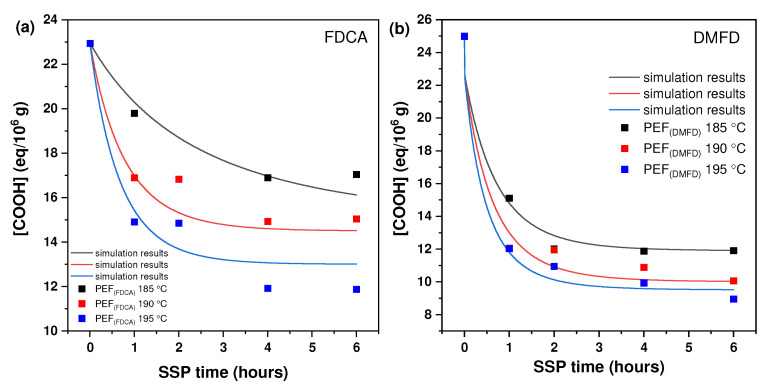
The concentration of carboxyl (−COOH) end group changes with time during the SSP of (**a**) PEF (FDCA) and (**b**) PEF (DMFD) at various temperatures. The continuous lines reflect the theoretical data collected from the kinetic model simulation.

Figure 2 shows the effect of reaction temperature and time during SSP on the carboxyl end-group concentration for PEF (FDCA) and PEF (DMFD) samples. The terminal carboxylic groups (–COOH) are the second variable that was utilized to track the effects of SSP time and temperature. The presence of carboxyl end groups is proof of the degradation reaction that happened during polycondensation step [32]. These formed carboxyl end groups that might react through esterification, which increases the molecular weight. From the results, it is understood that the concentration of the carboxyl end group decreased with an increase in SSP time and temperature in both PEF (FDCA) and PEF (DMFD) samples. The carboxyl end-group concentration was mostly dependent on time, whereas temperature had less of an effect on the variation.

The carboxyl group reduction rate was increased at 195 °C for both PEF (FDCA) and PEF (DMFD) samples, compared to 190 °C and 185 °C. The initial carboxyl end-group concentration of PEF (FDCA) 195 °C sample started at 22.95 eq/10^6^ g and reached 11.88 eq/10^6^ g after 6 h, whereas PEF (DMFD) 195 °C samples began at 25 eq/10^6^ g and reached 8.96 eq/10^6^ g after 6 h. Hence, it can be concluded that the carboxyl end-group concentration showed a consistent downward trend as the SSP time increased and a more significant reduction was observed in PEF (DMFD) samples compared to PEF (FDCA) samples.

**Figure 3:**
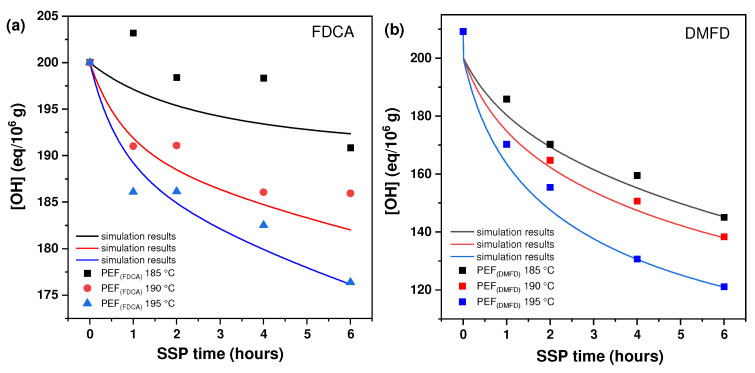
The concentration of hydroxyl (–OH) end group changes with time during the SSP of (**a**) PEF (FDCA) and (**b**) PEF (DMFD) at various temperatures. The continuous lines reflect the theoretical data collected from the kinetic model simulation.

Figure 3 displays the hydroxyl (–OH) end-group concentration of PEF (FDCA) and PEF (DMFD) samples. The (–OH) end-group concentration was the third variable that clarified how temperature and time affected SSP. As anticipated, and consistent with the previously described findings, the concentration of (–OH) end groups reduced over time. The PEF (FDCA) and PEF (DMFD) samples studied at 195 °C show the highest reduction compared to 185 °C and 190 °C. This rapid decrease in the (–OH) end-group concentration was in accordance with the [*ղ*] results. In comparison to FDCA, DMFD samples showed a faster and more consistent reduction trend as the SSP time increased. For example, the hydroxyl end-group concentration of PEF (FDCA) 195 °C samples started at 200 eq/10^6^ g and reduced to 176.37 eq/10^6^ g after 6 h of SSP, whereas PEF (DMFD) samples began at 209.24 eq/10^6^ g and decreased up to 121.18 eq/10^6^ g under the same conditions. Finally, during SSP of PEF (FDCA) and PEF (DMFD) polyesters, the mobility of the end groups was increased with respect to time and temperature, resulting in the chain extension of the polymers.

**Figure 4:**
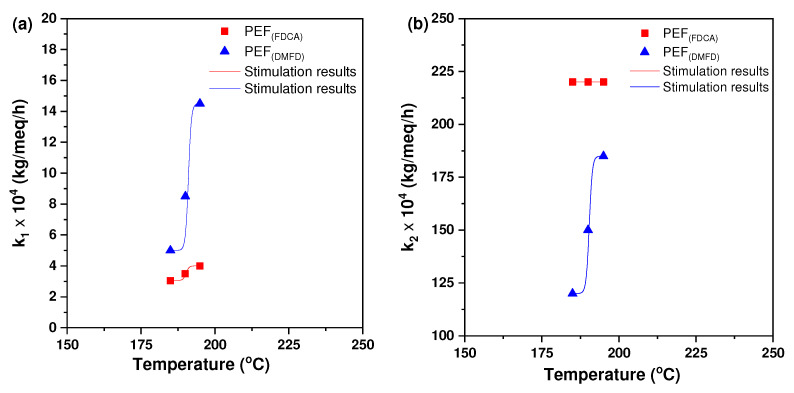
The calculated kinetic rate constants for the (**a**) polycondensation/transesterification (k_1_) and (**b**) esterification (k_2_) processes of PEF (FDCA) with PEF (DMFD) samples increased as a function of temperature.

The comparison between the fitted model curves and the experimental data appears in Figure 4. The values of the parameters found by fitting the experimental data are presented in Table 2. It was observed that the constant k_2_ was much larger than the constant k_1_. The larger values of k_2_ are compensated by the fact that the concentration of active carboxyls is much smaller than the one of active hydroxyls, so the two reactions (between chain-end hydroxyls and between chain-end hydroxyls and carboxyl’s) proceed in a comparable rate for a while until the elimination of chain-end carboxyl’s. The concentration of inactive species was reduced as the temperature increased, with the only exception being one of the hydroxyls for FDCA, where the inactive material remained almost constant with temperature.

The reaction constants increased with temperature, so the values of activation energy can be computed. By fitting an Arrhenius equation, it can be found that for DFMD, the reaction “1” between hydroxyls has activation energy Ε/R = 22,500 K (where R is the ideal gas constant) and the reaction “2” between hydroxyls and carboxyls has activation energy E/R = 9500 K. The corresponding values of the preexponential constant are 1.08∙10^18^ kg/meq/h and 1.22∙10^7^ kg/meq/h, respectively. Regarding FDCA, the activation energies for the reactions “1” and “2” were E/R = 5900 K and E/R = 0 K, respectively. The corresponding preexponential constants were 120 kg/meq/h and 0.022 kg/meq/h, respectively. The activation energy appeared to be much smaller for reaction “2” compared to reaction “1”. In addition, it appears to be much smaller for FDCA compared to DFMD. The zero value of activation energy of reaction “2” for FDCA implicated that the actual activation energy was small and could not be estimated with accuracy by the present experimental data.

### 4.2. Thermal Analysis of Polyesters After SSP

#### 4.2.1. DSC Analysis

**Figure 5:**
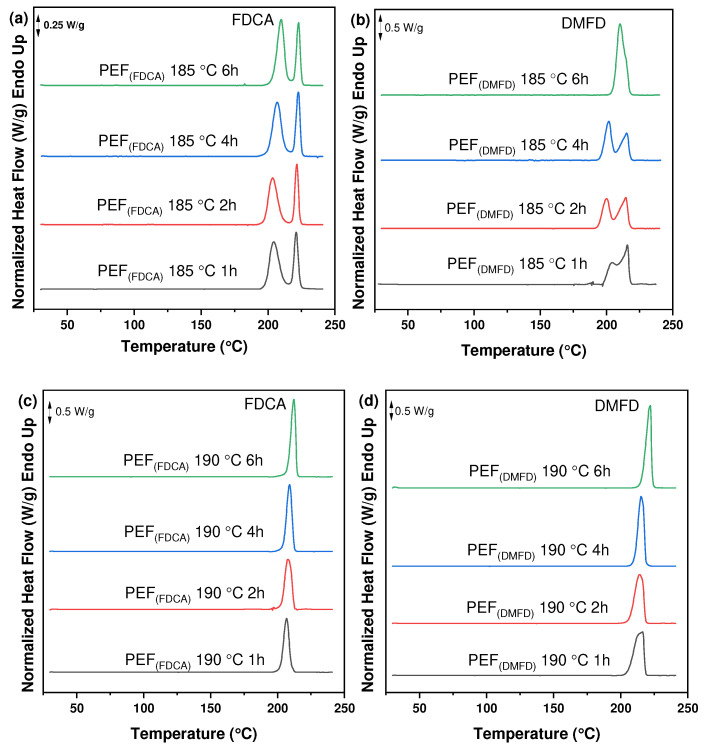

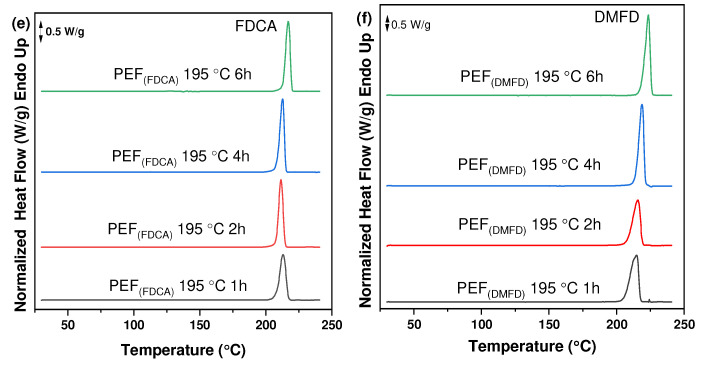
DSC thermograms of the PEF polyesters after SSP at 185 °C for (**a**) PEF (FDCA) and (**b**) PEF (DMFD), after SSP at 190 °C for (**c**) PEF (FDCA) and (**d**) PEF (DMFD), and after SSP at 195 °C for (**e**) PEF (FDCA) and (**f**) PEF (DMFD).

The thermal characteristics of PEF polyesters synthesized using FDCA and DMFD monomers were investigated using DSC thermograms. Figure 5a–f clearly illustrates that SSP time and temperature have a significant impact on the thermal properties of prepared PEF polyesters. The melting temperature and degree of crystallinity rose as the time and temperature of SSP increased, which is related to the rise in intrinsic viscosity and molecular weight (Table 1).

**Figure 6:**
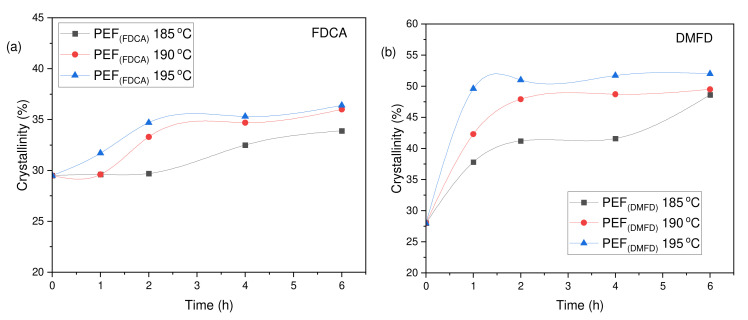
Evolution of the crystallinity degree (*X*_c_^a^ (%)) with respect to SSP time and temperature for (**a**) PEF (FDCA) and (**b**) PEF (DMFD) polyesters.

The degree of crystallinity (*X*_c_^a^ (%)) of the PEF polyesters was calculated using Equation (3) from the values of enthalpy (∆*H_m_*), displayed in Table 3. The evolution of the crystallinity of both PEF (FDCA) and PEF (DMFD) samples with respect to SSP time and temperature are displayed in Figure 6a,b, respectively. The PEF (FDCA) samples reached a maximum degree of crystallinity of 36.4%; on the other hand, the PEF (DMFD) samples reached a maximum of 52%. From the results, it is clear from the data that SSP occurs in the polymer’s amorphous regions. PEF (DMFD) samples’ rapid rise in molecular weight is associated with a lower degree of crystallinity. Because the undesirable byproducts were eliminated more quickly, the molecular weight increased. This claim is also supported by the values of $\bar{M_{n}}\;$/*ղ* found in the current investigation and the results of previous research on the SSP of PEF [15].

#### 4.2.2. XRD Analysis

**Figure 7:**
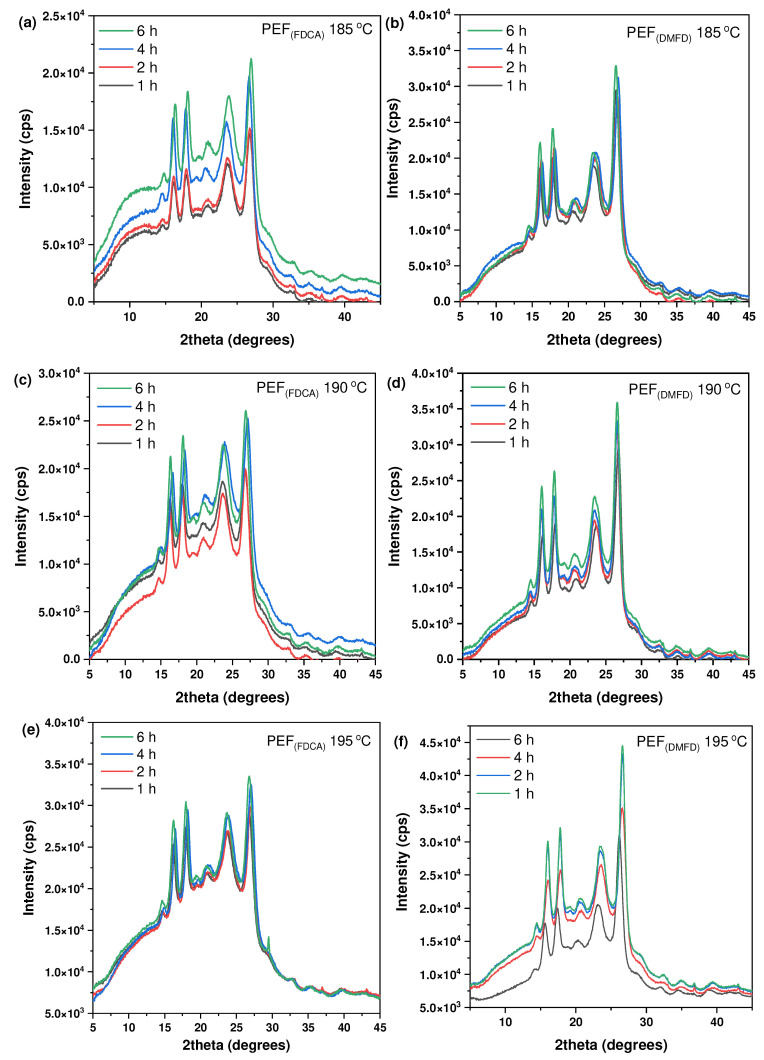
XRD patterns PEF polyesters after SSP at 185 °C for (**a**) PEF (FDCA) and (**b**) PEF (DMFD), after SSP at 190 °C for (**c**) PEF (FDCA) and (**d**) PEF (DMFD), and after SSP at 195 °C for (**e**) PEF (FDCA) and (**f**) PEF (DMFD).

The crystalline structure of the prepared PEF polyesters after SSP was investigated using XRD analysis. The resulting patterns of PEF (FDCA) and PEF (DMFD) samples are presented in Figure 7a–f. As observed, with respect to the increase in time and temperature, the crystallinity was increased in both the FDCA and DMFD samples. All the PEF polyesters exhibited four main diffraction peaks at 2θ ≈ 16.5°, 2θ ≈ 18°, 2θ ≈ 23°, and 2θ ≈ 26°, which indicated the influence of crystallization in samples after SSP [33]. However, the intensity of the peak in PEF (FDCA) samples was lower compared to the PEF (DMFD) samples. As reported in our previous work, FDCA samples have limited chain mobility, which causes a decrease in the crystallization rate. The higher DEG concentration (6%) in FDCA samples affected the crystallinity of polyesters due to the mobility of the ether block. Furthermore, decreasing DEG concentration (1–2%) in DMFD samples contributed to the increased crystallization rate [22,34].

**Figure 8:**
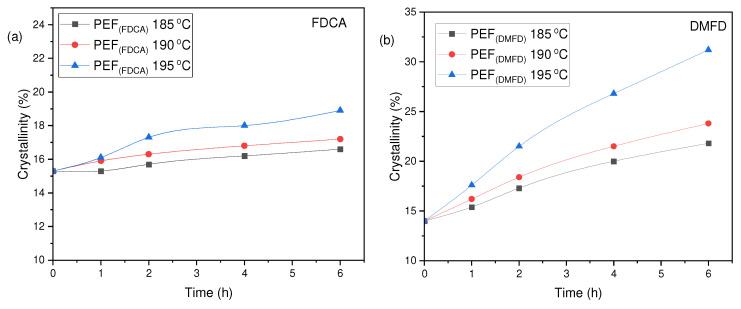
Evolution of the crystallinity degree (*X*_c_^b^ (%)) with respect to SSP time and temperature for (**a**) PEF (FDCA) and (**b**) PEF (DMFD) polyesters.

The degree of crystallinity (*X*_c_^b^) has been calculated using Equation (4) displayed in Table 4. From the results, it is observed that PEF (DMFD) samples show an increase in crystallinity with respect to SSP time and temperature compared to the PEF (FDCA) samples, as displayed in Figure 8a,b, respectively. PEF (FDCA) samples reached the highest crystallinity of 18.9%, whereas PEF (DMFD) samples reached the highest crystallinity of 31.2%. This finding shows that the SSP was performed in the amorphous regions of polymers and in agreement with the (*X*_c_^a^) and $\bar{M_{n}}$/*ղ* values obtained in this present study.

## 5. Conclusions

High-molecular-weight (0.43 dL/g) poly(ethylene 2,5-furan dicarboxylate) (PEF) polyesters were synthesized using 2,5-furan dicarboxylic acid (FDCA) and/or its derivative Dimethyl-2,5-Furan dicarboxylate (DMFD) monomers, followed by solid-state polymerization (SSP) conducted at different reaction times and temperatures. As expected, both PEF (FDCA) and PEF (DMFD) samples showed an increase in intrinsic viscosity [*ղ*] and number-average molecular weight ($\bar{M_{n}}$) with respect to SSP time and temperature. The SSP kinetics of PEF (FDCA) and PEF (DMFD) samples were investigated at different temperatures, 185 °C, 190 °C, and 195 °C, both experimentally and by simple kinetic modeling. The results indicated that the kinetic rate constant k_2_ was found to be significantly greater than the kinetic rate constant k_1_ due to the lower concentration of active carboxyls than that of active hydroxyls. The thermal analysis of both PEF polyester samples after SSP was performed by differential scanning calorimetry (DSC) technique revealed that the prepared PEF samples’ melting temperatures increased gradually in response to SSP temperature and reaction time. All SSP samples that were subjected to DSC and X-ray diffraction (XRD) analysis showed an increase in crystallinity. The PEF (DMFD) samples showed a rapid increase in crystallization and molecular weight due to their lower degree of crystallinity and high purity of monomer. The overall results showed that SSP occurs in the amorphous regions of the polymer, as anticipated for all SSP samples, and that the intrinsic viscosity and number-average molecular weight of prepared PEF polyester increased as the SSP temperature and time increased, while the concentration of carboxyl end groups decreased.

## Figures and Tables

**Table 1:** Intrinsic viscosity ([*ղ*], dL/g) and number-average molecular weights ($\bar{M_{n}}$.

Temperature (°C)	SSP Time (h)	PEF (FDCA)		PEF (DMFD)	
		**[*ղ*]**	$\bar{M_{n}}$	**[*ղ*]**	$\bar{M_{n}}$
as received	0	0.43	8969 (49)	0.41	6822 (37)
185	1	0.43	8969 (49)	0.46	9950 (54)
	2	0.44	9292 (50)	0.49	10,967 (59)
	4	0.44	9292 (50)	0.51	11,664 (63)
	6	0.45	9619 (52)	0.54	12,737 (69)
190	1	0.45	9619 (52)	0.49	10,967 (59)
	2	0.45	9619 (52)	0.50	11,313 (63)
	4	0.46	9950 (54)	0.53	12,376 (67)
	6	0.46	9950 (54)	0.56	13,471 (73)
195	1	0.46	9950 (54)	0.49	10,967 (59)
	2	0.46	9950 (54)	0.52	12,018 (65)
	4	0.47	10,285 (56)	0.58	14,219 (77)
	6	0.48	10,624 (57)	0.61	15,367 (83)

**Table 2:** Parameter values resulted by fitting the simplified model to the experimental data of chain-end hydroxyl and carboxyl evolution.

Sample	k_1_ (kg/meq/h)	k_2_ (kg/meq/h)	[OH]_i_ (meq/kg)	[COOH]_i_ (meq/kg)
FDCA				
185 °C	0.000305	0.022	80	15
190 °C	0.00035	0.022	90	14.5
195 °C	0.0004	0.022	85	13
DFMD				
185 °C	0.0005	0.012	180	12
190 °C	0.00085	0.015	140	10
195 °C	0.00145	0.0185	130	9.5

**Table 3:** Crystallinity degree (*X*_c_^a^ (%)) of PEF (FDCA) and PEF (DMFD) samples after SSP calculated from DSC using melting enthalpy (∆*H_m_*).

SSP Temperature (°C)	SSP Time (h)	PEF (FDCA) (*X*_c_^a^ (%))	PEF (DMFD) (*X*_c_^a^ (%))
As received	0	29.5	28
185	1	29.6	37.8
	2	29.7	41.2
	4	32.5	41.6
	6	33.9	48.6
190	1	29.6	42.3
	2	33.3	47.9
	4	34.7	48.7
	6	36	49.5
195	1	31.7	49.6
	2	34.7	51
	4	35.3	51.7
	6	36.4	52

**Table 4:** Crystallinity degree (*X*_c_^b^ (%)) of PEF (FDCA) and PEF (DMFD) samples after SSP calculated from XRD thermograms.

SSP Temperature (°C)	SSP Time (h)	PEF (FDCA) (*X*_c_^b^ (%))	PEF (DMFD) (*X*_c_^b^ (%))
As received	0	15.3	14
185	1	15.3	15.4
	2	15.7	17.3
	4	16.2	20
	6	16.6	21.8
190	1	15.9	16.2
	2	16.3	17.4
	4	16.8	21.5
	6	17.2	23.8
195	1	16.1	17.6
	2	17.3	21.5
	4	18	26.8
	6	18.9	31.2

## Data Availability

Data are contained within the article.

## Abbreviations

The following abbreviations are used in the manuscript.

PEF	Poly(ethylene 2,5-furan dicarboxylate)
DMFD	Dimethyl-2,5 furandicarboxylate
FDCA	2,5-furan dicarboxylic acid
XRD	X-ray diffraction
PET	Poly(ethylene terephthalate)
GHG	Greenhouse gas
LCA	Life cycle assessment
EG	Ethylene glycol
SSP	Solid-state polymerization
PPF	Poly(propylene furanoate
PBF	Poly(butylene furanoate
DSC	Differential scanning calorimetry
WAXD	Wide Angle X-ray Diffraction Patterns
DGO	Diguaiacyl oxalate
